# Cybersecurity Risks in a Pandemic

**DOI:** 10.2196/23692

**Published:** 2020-09-17

**Authors:** Christina Meilee Williams, Rahul Chaturvedi, Krishnan Chakravarthy

**Affiliations:** 1 Trinity College of Arts & Sciences Duke University Durham, NC United States; 2 School of Medicine University of California, San Diego La Jolla, CA United States; 3 Department of Anesthesiology University of California, San Diego La Jolla, CA United States

**Keywords:** cybersecurity, pandemic, COVID-19, SARS-CoV-2, risk, privacy, hack, patient data

## Abstract

Cybersecurity threats are estimated to cost the world US $6 trillion a year by 2021, and the number of attacks has increased five-fold after COVID-19. Although there is substantial literature on the threats technological vulnerabilities have on the health care industry, less research exists on how pandemics like COVID-19 are opportunistic for cybercriminals. This paper outlines why cyberattacks have been particularly problematic during COVID-19 and ways that health care industries can better protect patient data. The Office for Civil Rights has loosened enforcement of the Health Insurance Portability and Accountability Act, which, although useful in using new platforms like Zoom, has also loosened physical and technical safeguards to cyberattacks. This is especially problematic given that 90% of health care providers had already encountered data breaches. Companies must implement well-defined software upgrade procedures, should use secure networks like virtual local area networks, and conduct regular penetration tests of their systems. By understanding factors that make individuals, health care organizations, and employers more susceptible to cyberattacks, we can better prepare for the next pandemic.

As society has become increasingly technology dependent, it has also become increasingly vulnerable to cybercrime. Cybersecurity threats are expected to cost the world US $6 trillion a year by 2021, doubling from US $3 trillion dollars in 2015 [1]. This is particularly concerning for the health care industry, as cyberattacks are the leading cause of health security breaches [2]. Since 2016, the health care industry has been the victim of more cybersecurity attacks than even the financial industry [3]. Although there is substantial literature on the threats technological vulnerabilities have on the health care industry, less research exists on how pandemics like COVID-19 are opportunistic for cybercriminals. In this paper, we provide a review of the literature on cybersecurity issues surrounding health care and discuss possible solutions to mitigate data breaches.

One of the primary reasons cybercriminals thrive during pandemics is because heightened emotional states like fear make victims more susceptible to falling for scams [4]. According to the World Health Organization (WHO), the number of cyberattacks launched has increased five-fold during the COVID-19 pandemic [5]. A similar phenomenon was seen in 2005 after Hurricane Katrina, where thousands of fraudulent websites appeared soliciting fake donations and offering false government relief [6]. Cybercriminals often pretend to be credited and trusted organizations like the WHO and, therefore, exploit individual feelings of vulnerability in the uncertain times of a pandemic. 

Additionally, health care organizations become prime targets during health crises. The use of telemedicine has proven vital to helping many patients during pandemics such as the COVID-19 crisis, especially as traditional in-person visits have become increasingly inaccessible. For example, New York University saw a 4330% increase in nonurgent virtual visits after the outbreak of COVID-19 [7]. The Office for Civil Rights has loosened enforcement of the Health Insurance Portability and Accountability Act (HIPAA), which, although useful in opening up new platforms for care like Zoom, Skype, and FaceTime, has loosened physical and technical safeguards to cyberattacks [2,8]. This is especially problematic given that 90% of health care providers had already encountered data breaches in the past with these safeguards [2]. There is also a significant positive correlation between workload and the probability a health care worker will open a phishing email, which is particularly problematic in that, during pandemics, workloads can be at an all-time high [9].

Another potential problem for health care systems is the outbreak of ransom-motivated attacks. For example, the University of California, San Francisco (UCSF) was hacked by the cybercrime group “Netwalker,” who demanded payment in exchange for not releasing confidential information. Out of fear of the consequences of this information’s release, UCSF paid the group US $1.14 million [10]. The same group also took over the Champaign Urbana Public Health District website. Similarly, the Hollywood Presbyterian Medical Center in Los Angeles paid US $17,000 to get a decryption key to regain access to their hospital system. Although they regained access, they lost 10 days of revenue and likely took a hit to their reputation [2]. Unfortunately, however, complying with the demands of the cybercriminal may in fact be the most cost-effective solution, as a successful cyberattack costs an average of US $3.7 million to recover from [2]. Additionally, failure to comply can pose a serious threat to patient safety.

Access to patient records is a gold mine for cybercriminals, as they often contain information like date of birth, insurance and health provider information, as well as genetic and health data—information that cannot be easily altered, unlike the case of a credit card being stolen [3]. This information is particularly lucrative for hackers because a patient’s health information can be sold for 10-20 times more than the amount for credit card information or even their social security number on the dark web. 

Leak of this information can also compromise the physician-patient relationship. For instance, electronic medical record breaches could make patients less likely to disclose more private aspects of their medical history, which has the potential to impact their quality of care [11]. Furthermore, the longer a health care provider’s network is down, the longer those health care workers lack access to information critical to a patient’s care, like comorbidities, blood type, and allergies, in times of crisis [3]. The cost both financially and in terms of reputation and patient safety can cripple already strained hospital operations.

One additional avenue of attack presents itself as a result of the increase in the number of health care workers working from home during a pandemic like COVID-19. In the attempt to transition employees to a work-from-home setup as quickly as possible, many employers fail to consider the potential security threats these new setups create. For instance, in the hospital or office, employees may be using secure internal computer systems and updated computers, but at home, the same employees could be using insecure or outdated devices that are more vulnerable to attack [4]. Although many hospitals opted to use the Zoom platform because they view it as HIPAA-compliant, easy for both providers and patients to use, and cost-effective with medical videoconferencing accounts costing only US $200 a month, hacking of Zoom meetings has been a significant threat. Services like Zoom currently do not offer end-to-end encryption, making it not truly HIPAA-compliant, even though the Department of Health and Human Services Office for Civil Rights has relaxed enforcement of HIPAA’s privacy rule during the COVID-19 pandemic [12]. 

Although the issue of how to safely administer health care during a pandemic is a complex one, it is clear that increased awareness is needed concerning the potential cyberthreats that pandemics exacerbate. Awareness of these threats can help hospitals and their employees protect themselves and their patients from these vulnerabilities. For instance, being aware that hackers develop phishing scams containing buzzwords during a pandemic, like “WHO,” “vaccine,” or “donation,” can be an essential step in reviewing and flagging such emails, thereby tightening security by the information technology (IT) departments. One technique that can be employed is to have hospital IT departments send out fake phishing emails to their employees and to require training for those who failed to report the phishing attempt [13]. At the very least, this process can raise awareness among employees about cybersecurity concerns. Companies should also have well-defined software upgrade procedures, should use secure networks like virtual local area networks, and conduct regular penetration tests of their systems [2]. Hospitals need to more closely monitor administrative privileges, as the majority of large scale attacks began with a compromised account like that of a third-party provider, as seen in the case of the Hancock Regional Hospital in January 2018 [3]. By monitoring the log activity of user accounts and revoking account access when no longer needed, and employing techniques such as multifactor authentication, hospitals can better protect their IT infrastructure [3].

By understanding the factors that make individuals, health care organizations, and employers more susceptible to cyberattacks, we can better prepare for the next pandemic.

## Abbreviations

HIPAA: Health Insurance Portability and Accountability Act
IT: information technology
UCSF: University of California, San Francisco
WHO: World Health Organization
